# Supplementary material for: Structures of TASK-1 and TASK-3 K2P channels provide insight into their gating and dysfunction in disease
Source: Structure. Author manuscript; Available in PMC 2025 Jul 17. (PMC7617898; doi:10.1016/j.str.2024.11.005)
Supplement: Supplementary Materials [file EMS206718-suppement-Supplementary_Materials.pdf]

Supplementary Information:

Table S1. Cryo-EM data collection, refinement and validation statistics, related to Figures 1-3.

	**TASK-3**	**TASK-3 (G236R)**	**TASK-1**
	**9G9V EMD-51158**	**9G9W EMD-51159**	**9G9X EMD-51160**
**Data collection and processing**			
Magnification	105,000	105,000	105,000
Voltage (kV)	300	300	300
Electron exposure ($e^{-}/Å^2$)	39.56	40.877	41.173
Defocus range (µm)	-1.2 to -2.7	-0.75 to -2.25	-1.25 to -2.5
Pixel size (Å)	0.832	0.832	0.832
Symmetry imposed	C2	C2	C2
Initial particle images (no.)	12,215,148	8,941,912	8,116,956
Final particle images (no.)	93,760	124,588	117,627
Map resolution (Å)	3.32	2.48	3.13
FSC threshold	0.143	0.143	0.143
**Refinement**			
Initial model used	Manually	Manually	Crystal structure (6RV2)
Non hydrogen atoms	4073	4089	3955
Protein residues	522	516	488
Average *B* factors (Å2)			
Protein	142.54	104.76	119.5
Ligand	145.73	115.31	123.48
r.m.s.d.			
Bond lengths (Å)	0.008	0.008	0.008
Bond angles (°)	1.140	1.111	1.143
**Validation**			
MolProbity score	0.93	0.82	0.80
Clashscore	1.73	1.11	1.26
Poor rotamers (%)	0.53	0.00	0.00
**Ramachandran plot**			
Favoured (%)	98.46	99.61	99.17
Allowed (%)	1.54	0.39	0.83
Outliers (%)	0.00	0.00	0.00

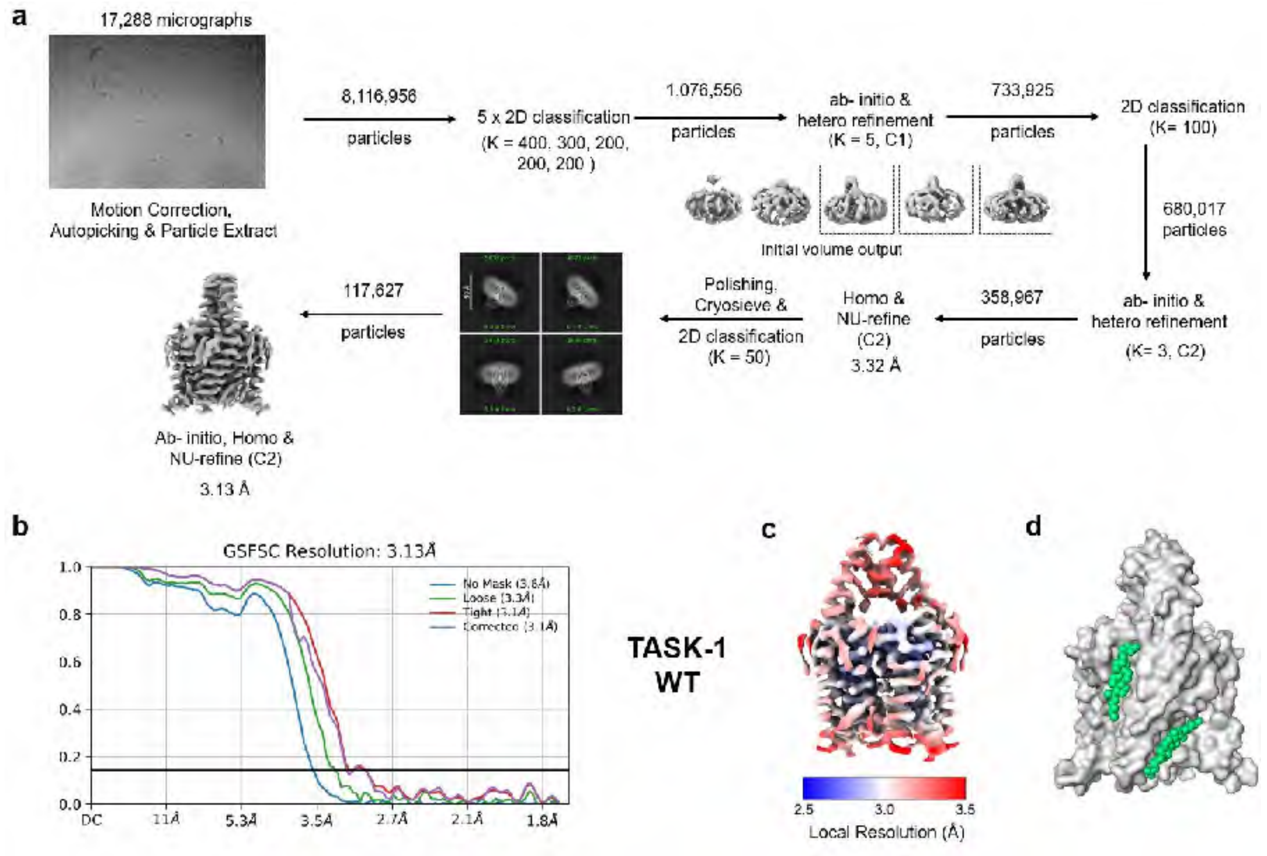

Supplementary Figure S1. Cryo-EM processing workflow for TASK-1 showing the local resolution map, related to Figure 1. **a**, Image processing workflow for TASK-1. **b**, Gold-standard FSC curve used for global-resolution estimates within cryoSPARC. **c**, Local-resolution of reconstructed map as determined within cryoSPARC. **d**. Surface representation of TASK-1 indicated the relative position of CHS resolved in the structure as green VdW spheres.

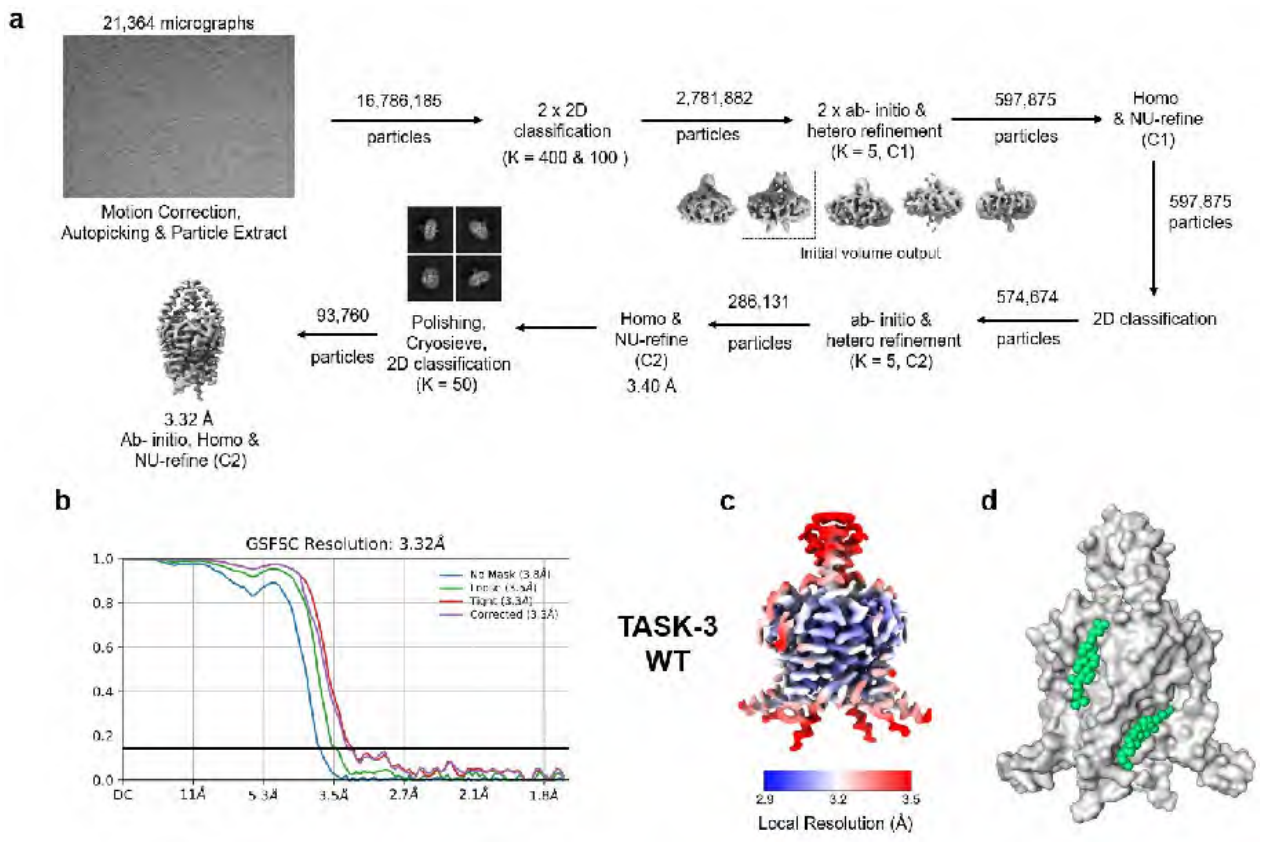

Supplementary Figure S2. Cryo-EM processing workflow for TASK-3 showing the local resolution map, related to Figure 2. **a**, Image processing workflow for TASK-3. **b**, Gold-standard FSC curve used for global-resolution estimates within cryoSPARC. **c**, Local-resolution of reconstructed map as determined within cryoSPARC. **d**. Surface representation indicating the relative position of CHS resolved in the structure as green VdW spheres.

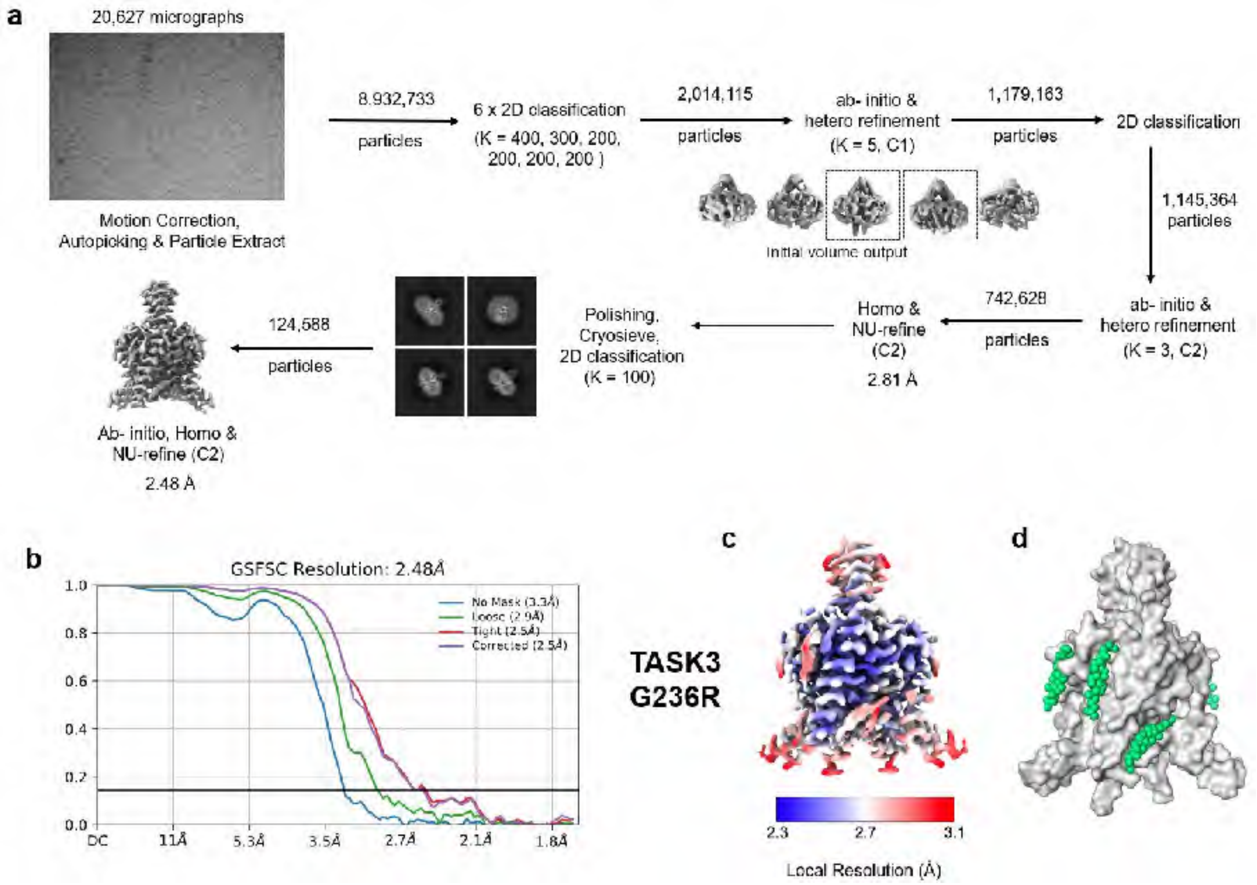

Supplementary Figure S3. Cryo-EM processing workflow for G236R TASK-3 showing the local resolution map, related to Figure 3. **a**, Image processing workflow for G236R TASK-3. **b**, Gold-standard FSC curve used for global-resolution estimates within cryoSPARC. **c**, Local-resolution of reconstructed map as determined within cryoSPARC. **d**. Surface representation indicating the relative position of CHS resolved in the structure as green VdW spheres. The higher resolution of this structure reveals three CHS molecules.
